# Comparing the Data Quality of Global Positioning System Devices and Mobile Phones for Assessing Relationships Between Place, Mobility, and Health: Field Study

**DOI:** 10.2196/mhealth.9771

**Published:** 2018-08-13

**Authors:** Robert Goodspeed, Xiang Yan, Jean Hardy, VG Vinod Vydiswaran, Veronica J Berrocal, Philippa Clarke, Daniel M Romero, Iris N Gomez-Lopez, Tiffany Veinot

**Affiliations:** ^1^ Urban and Regional Planning Program Taubman College of Architecture and Urban Planning University of Michigan Ann Arbor, MI United States; ^2^ School of Information University of Michigan Ann Arbor, MI United States; ^3^ Department of Learning Health Sciences Medical School University of Michigan Ann Arbor, MI United States; ^4^ Department of Biostatistics School of Public Health University of Michigan Ann Arbor, MI United States; ^5^ Department of Epidemiology School of Public Health University of Michigan Ann Arbor, MI United States; ^6^ Institute for Social Research University of Michigan Ann Arbor, MI United States; ^7^ Department of Health Behavior and Health Education School of Public Health University of Michigan Ann Arbor, MI United States

**Keywords:** urban population, spatial behavior, mobile phone, environment and public health, data accuracy

## Abstract

**Background:**

Mobile devices are increasingly used to collect location-based information from individuals about their physical activities, dietary intake, environmental exposures, and mental well-being. Such research, which typically uses wearable devices or mobile phones to track location, benefits from the growing availability of fine-grained data regarding human mobility. However, little is known about the comparative geospatial accuracy of such devices.

**Objective:**

In this study, we compared the data quality of location information collected from two mobile devices that determine location in different ways—a global positioning system (GPS) watch and a mobile phone with Google’s Location History feature enabled.

**Methods:**

A total of 21 chronically ill participants carried both devices, which generated digital traces of locations, for 28 days. A mobile phone–based brief ecological momentary assessment (EMA) survey asked participants to manually report their location at 4 random times throughout each day. Participants also took part in qualitative interviews and completed surveys twice during the study period in which they reviewed recent mobile phone and watch trace data to compare the devices’ trace data with their memory of their activities on those days. Trace data from the devices were compared on the basis of (1) missing data days, (2) reasons for missing data, (3) distance between the route data collected for matching day and the associated EMA survey locations, and (4) activity space total area and density surfaces.

**Results:**

The watch resulted in a much higher proportion of missing data days (*P*<.001), with missing data explained by technical differences between the devices as well as participant behaviors. The mobile phone was significantly more accurate in detecting home locations (*P*=.004) and marginally more accurate (*P*=.07) for all types of locations combined. The watch data resulted in a smaller activity space area and more accurately recorded outdoor travel and recreation.

**Conclusions:**

The most suitable mobile device for location-based health research depends on the particular study objectives. Furthermore, data generated from mobile devices, such as GPS phones and smartwatches, require careful analysis to ensure quality and completeness. Studies that seek precise measurement of outdoor activity and travel, such as measuring outdoor physical activity or exposure to localized environmental hazards, would benefit from the use of GPS devices. Conversely, studies that aim to account for time within buildings at home or work, or those that document visits to particular places (such as supermarkets, medical facilities, or fast food restaurants), would benefit from the greater precision demonstrated by the mobile phone in recording indoor activities.

## Introduction

### Background

Significant relationships between health and the places in which we live and work are now widely acknowledged, with associations having been found for health behaviors ranging from diet to physical activity to the use of health care services [1,2]. In addition, location-based data acquired from mobile devices can be used to assess physical activity [3], exposures to hazardous substances [4], and symptoms of mental health conditions such as depression [5-8]. Accordingly, there is growing interest in the use of location-tracking devices as a data collection tool for health research.

Researchers investigating relationships between health, place, and mobility require location-tracking devices, which are both acceptable to users and accurate. To date, a variety of pilot and feasibility studies have examined user acceptance of devices such as wearable activity trackers [9,10], dedicated global positioning system (GPS) devices [11], and GPS-enabled mobile phones [12,13]. Although this research generally shows reasonable acceptance among varied user groups, there remain gaps in understanding the spatial accuracy of each of these devices, particularly those that are available on the consumer market and thus could facilitate population-level research. Therefore, building on prior health informatics research examining the accuracy of other types of devices used in research [14,15], we compared the accuracy of two widely available location-tracking devices that determine location using two different technical approaches (described further below).

One of the key approaches adopted in health research that uses location-tracking data focuses on characterizing the spaces in which participants typically spend their time; this is typically used to overcome the limitations of determining participant location solely based on the place of residence [16]. This is important as recent research has shown that environmental characteristics of such nonresidential places are associated with health-related outcomes, such as self-rated health [17] and dietary intake [18-20]. Therefore, a growing number of studies on health, place, and mobility are using an analytical approach that involves constructing an *activity space* for each participant (eg, [21-24]). This concept, proposed by space-time geographers, describes the portion of the environment actually used by an individual to fulfill activities and travel between locations [25-27].

Activity space construction requires detailed spatial information from participants, ideally records of travel through the environment collected in real time. Accordingly, researchers have used GPS devices to study the relationship between the environment and physical activity [28] and food environments and eating [29]. However, GPS devices pose a set of well-known research challenges: commercially available devices can have limited battery life and nonintuitive interfaces, are often physically bulky, and lose satellite signals indoors or in dense urban environments [30-32]. Mobile phones offer one potential alternative to dedicated GPS devices; they provide integrated location services through a combination of GPS, cellular tower triangulation, and geolocation of Wi-Fi networks. As mobile phone location information is acquired through multiple methods, these devices work in a variety of physical contexts and may result in more accurate data about activities taking place within and close to buildings. In addition, mobile phones may have other benefits such as greater acceptability by study participants which, in turn, may reduce data loss [13]. Although dedicated apps can collect location data, Android devices can record location information automatically via the integrated Google Location History feature, making the collection and analysis of mobility data feasible at the population level. Tracking studies such as this one raise important ethical concerns, such as those around the privacy of the data collected [33]. As described further below, we took typical steps to obtain informed consent and protect the privacy of the data collected. Although privacy is not the primary focus of this study, we briefly comment on this issue in the Discussion.

### Objectives

In this study, we evaluated the quality of location data generated from mobile phones and a wearable GPS device, providing a novel direct comparison. The three specific comparative aspects of the mobile devices that we examined in this study were (1) data loss experienced, (2) accuracy of the gathered data, and (3) activity spaces generated using the data. We focused on people with diabetes, hypertension, and chronic kidney disease as spatially sensitive health behaviors such as physical activity, diet, medication, and health care appointment adherence are important drivers of outcomes in people with these conditions (eg, [34-36]).

## Methods

### Participant Recruitment

This study was conducted as part of a larger research project focused on *big data* methods for characterizing the relationship between place of residence and health, with a focus on new data sources such as social media [37,38] and location tracking using mobile devices. This study focused on diabetes, hypertension, and kidney disease, as these conditions often co-occur [39] and have outcomes that are influenced by health behaviors such as eating, physical activity, and treatment adherence [40-42]. Moreover, prior research has shown that these behaviors are related to neighborhood contextual features such as the food environment, physical activity resources, and local health care services [43-47]. Accordingly, location-tracking devices hold particular promise for studying the relationship between place, health behavior, and health outcomes in this patient group. Participants were recruited through a university portal for research participants at the University of Michigan. Eligible participants were 18 years or older and self-reported at least one of these conditions.

### Device Deployment

Participants were provided with two consumer-oriented location-tracking mobile devices to carry for 28 days, although some kept the devices for longer based on our ability to schedule a final interview with them. We selected two mobile devices for this study—a GPS-enabled device and a mobile phone with location tracking enabled. The GPS device used was a Garmin fēnix 2 GPS Watch (henceforth referred to as the watch), whereas the mobile phone was a Samsung Galaxy S5 (henceforth referred to as the phone). As the phone is an Android device, it has an integrated Google Location History feature that was enabled to track locations as part of the study. These devices were chosen to represent consumer-oriented technologies that are widely available at reasonable price-points (US $399 for the watch and US $650 for the mobile phone without a contract) and that use the two prevalent methods of determining user location: GPS satellites as opposed to mobile phones’ use of a combination of cellular towers, Wi-Fi networks, and GPS triangulation.

Participants were asked to keep the devices switched on for at least 8 hours daily, ideally from the time they awoke until they went to bed. Each device was enabled to capture digital traces of participant location. The watch was set to the *Smart* default-sampling rate, which records data every 4 to 7 seconds. According to product forums, the sampling rate for Location History is variable and depends on factors such as the strength of available signals, battery strength, and whether the Google Maps app is running in the foreground or background. They were asked to keep the devices charged and synchronize data from the watch to the mobile phone every day. Participants were trained in the use of the devices and were given instruction cards as a follow-up reminder. They were also given a phone number for the technical support provided by the research team, and the devices were returned at the end of the study. Participants earned US $3.57 for each day of data they uploaded, up to a maximum of US $100 for the full 28 days. The study received ethical approval from the Health Sciences and Behavioral Sciences Institutional Review Board at the University of Michigan (HUM00098270).

### Data Collection Methods

In addition to the spatial information, a brief ecological momentary assessment (EMA) survey configured using the Personal Analytics Companion (PACO) software for Android phones presented two free-text survey questions to participants at 4 random times in a given day: “where are you?” and “what are you doing?” and prompted participants to take a photograph of their location using the mobile phone. Participants also took part in qualitative interviews and completed surveys twice during the study period—once after the first 14 days and then at the end of the 28-day period. In these two interviews, participants were asked about their experience using the devices and to review the phone and watch trace data on Web-based platforms designed for this purpose (Google Maps and the online website, Garmin Connect) for the 2-4 most recent days (the range depended on whether data were missing for recent days) and to compare the data with their memory of their activities on those days. Data were collected from summer to fall of 2015.

### Analysis Methods

The analyses presented here rely on three data sources: (1) digital location information logged by each device, (2) results of the brief EMA survey that inquired about participants’ locations and activities, and (3) interview results concerning reasons for data loss. The phone data resulted in a line representing travel routes, with selected points recorded along the route corresponding to destinations. On the other hand, the watch data consisted of a route and regular waypoints. Due to this difference, only the route (or trace) information from each device was used in this analysis. Points contained in the Keyhole Markup Language files that are exported from the Google Location History service are named according to the assumed mode of travel (eg, “Walking,” “Driving”) or specific destination (eg, “Olson Park”) assigned to the user by Google’s software. In addition, additional information such as raw data can be viewed, but not downloaded, on the Location History website. Either may be useful for researchers. Without waypoints for the phone data, standard smoothing techniques could not be applied to the phone data.

#### Missing Data Analysis

Although participants were asked to carry both devices for 28 days, the number of days in which they acquired 8 hours of data varied. Data completeness was evaluated by calculating the proportion of missing data days, defined as the number of days in which the participant did not collect 8 hours of data divided by the participant’s number of days in the study. As an additional measure of the amount of data collected, the number of days where any spatial information was collected was also reported.

The interview data containing user evaluations of the accuracy of the data contained user reflections on reasons for missing or incorrect data, which were coded using structural coding [48]. These coded data were compared with trace data for each participant for whom there was missing data, to identify the reasons for the specific missing data episodes. These reasons were then grouped into descriptive categories [48].

#### Geographic Information System Data Procedures

As described, the EMA surveys included a free-text field for the participants’ current location. Among the survey responses, 30.4% could be converted into a street address, including the terms “home” and “work,” or another identifiable location. Other locations included a park or campsite, business, theater, home of friend, health care organization, library, church, and gym. In other cases, respondents either provided no response to the other surveys or responses which could not be mapped. This resulted in 1375 geocoded destination points. Among these, 461 were collected on days in which spatial information was available from both the devices. The distance between the route data collected for that day and the associated survey locations was then computed using the spatial join tool in ArcGIS Desktop 10.3.1. The spatial join function was run using the “closest” option, which records the nearest location feature from either the phone or watch data to the point mapped from the PACO survey responses. The mean distances were then computed for each location category and for all locations combined.

#### Activity Space Analysis

Multiple methods have been proposed for converting raw spatial information into a representation of an activity space [22], such as the use of Census Tracts [23,49], the characteristics of known destinations [24], or constructing standard deviational ellipses from location information [50,51]. Tailored approaches include *travel time polygons* describing an area of potential travel, *road network buffers* of routes to regular destinations, or daily path areas constructed from actual routes taken [18,52]. We selected the daily path method because it utilizes the detailed route information collected from the devices and results in a representation of actual (vs potential) travel during the study period.

Therefore, an activity space was constructed using the daily path area method, which buffers digital location traces with buffer distance depending on the geographic context and study aims [18,22]. The activity space analysis was conducted for comparison days only, where data were available from both devices. The average number of days per participant was 19.2, with a range of 5 to 33. A buffer was applied to the data for those days. We used 400 m as the buffer distance because it can be considered as the minimum walking distance to reach a destination in an urban environment [53,54]. The buffer was then clipped to the participant’s home county to exclude nonroutine activities such as vacation travel. The total area of the resulting polygon was then computed. No changes or corrections to either data sources were made for this analysis, except for the removal of one obviously errant line from participant 14’s phone data, described further below.

In the activity space analyses, a *kernel density* function can be used to calculate a continuous surface from a set of known locations [55]. In a geographic information system, a kernel density function calculates a magnitude-per-unit area from a point or line feature using a kernel function to fit a smoothly tapered surface. The surface resulting from this analysis can be interpreted as a probability surface similar to that presented in the study by Downs et al [56]. We used this approach because unlike the buffering method, a kernel density function takes into account multiple traces in the same area, weighing them differently depending on the shape of the density function employed and the kernel density function bandwidth. In addition, unlike the buffer that delineates a specific area, the kernel density results in a continuous representation. As our purpose was to compare the data obtained from the 2 devices, the output of this analysis shows the difference between the density maps for each device. The method included the following steps: (1) clip the trace data to the participant’s home county, (2) compute the kernel density with a search radius of 400 m, (3) rescale the values in the density surface resulting from the phone and watch data so that they total to 1, and (4) compute the difference between the density surfaces.

## Results

### Characteristics of Participants

As shown in Table 1 below, 57% (12/21) of participants were women, and the mean participant age was 52.4 (SD 11.36) years, with a range of 33 to 74. Majority of the participants (17/21, 81%) had a bachelor’s degree or higher and just over half were employed. Two-thirds of the population were white and about one-third had another racial or ethnic identity. In this sample of chronically ill participants, 76% (16/21) had hypertension, 62% (13/21) had diabetes, and 19% (4/21) had chronic kidney disease.

### Missing Data Analysis

As shown in Table 2, the watch resulted in a much higher proportion of missing data days, primarily because of a large number of days on which data were collected for less than 8 hours (Z=3.920, *P*<.001). However, considering all days with any amount of valid trace data, the phone resulted in only slightly more data than the watch (*t*_20_=0.460, *P*=.65).

Interviews with the study participants revealed that the differences in missing data were explained by technical differences between the devices as well as participant behaviors. Explanations for these differences revealed in the interviews included the following: participants forgetting to initiate data collection at the beginning of the day on their GPS watch, the GPS watch not being able to locate satellites in certain buildings, removing the watch during activities participants perceived to be unsafe for the watch (eg, sports and kitchen work), and troubles syncing the watch data to the mobile phone so that it could be recorded.

### Spatial Accuracy Analysis

The mean distance from the points obtained from self-reported participant location based on the EMA survey data and the spatial data from each device was lower for the phone than for the watch overall and for each of the four categories considered (Table 3). The destination category with the smallest distance was home, followed by business. The two devices yielded marginally significantly different mean distances for all points (*P*=.07). When stratified by specific categories, the only statistically significant difference between the two devices has been found for home locations (*P*=.004).

### Activity Space Analysis

The resulting daily path area from the buffer analysis from both devices was much larger for the phone than for the watch. As shown in Table 4, the average activity space for the phone was 18,084.74 ha, whereas it was 10,494.57 ha for the watch. A two-sample *t* test with unequal variances found the difference is statistically significant at the 99% level (*t*_20_=3.164, *P*=.003). On average, the watch showed 42.0% fewer hectares in participants’ activity spaces.

**Table 1:** Participant demographics and technology ownership (N=21).

Characteristics		n (%)
**Gender**		
	Female	12 (57)
	Male	9 (43)
**Race^a^**		
	Black or African American	5 (24)
	White or European American	17 (81)
	Native American or Native Hawaiian or Pacific Islander	1 (5)
	Other	1 (5)
**Ethnicity**		
	Hispanic or Latino	3 (14)
	Non-Hispanic or Latino	18 (86)
**Education**		
	Some college	3 (14)
	Associate’s degree	1 (5)
	Bachelor’s degree	6 (29)
	Master’s degree or PhD	8 (38)
	Professional degree (eg, JD and MD)	3 (14)
**Employment^a^**		
	Full-time employment (30+ hours per week)	8 (38)
	Part-time employment (<30 hours per week)	3 (14)
	Student	2 (9)
	Unemployed	4 (19)
	Disabled	3 (14)
	Retired	4 (19)
**Health conditions^a^**		
	Hypertension	16 (76)
	Diabetes	13 (62)
	Chronic kidney disease	4 (19)

^a^More than one response possible.

**Table 2:** Missing data analysis results.

Measure	Phone	Watch	Z value	*t* value (*df*)	*P* value
Proportion of missing data days (<8 hours)	0.03	0.54	3.920	N/A^a^	<.001
Mean valid data days	23.5	22.2	N/A	0.460 (20)	.65

^a^N/A: not applicable.

**Table 3:** Spatial accuracy analysis.

Categories	Phone distance (m), mean (SD)	Watch distance (m), mean (SD)	Comparison		
			N	*t* value (*df*)	*P* value
Home	24.6 (38.6)	106.2 (576.9)	352	−2.649 (351)	.004
Work	2318.0 (2982.1)	2831.9 (3821.7)	81	−0.954 (80)	.17
Business	694.3 (1484.5)	804.2 (1549.4)	19	−0.315 (18)	.38
Other	360.4 (493.4)	1240.7 (2778.8)	9	−0.936 (8)	.19
All	461.7 (1547.1)	636.0 (2023.5)	461	−1.470 (460)	.07

**Table 4:** Activity space comparison from travel route buffer.

Participant ID	Comparison days	Phone area (ha^a^)	Watch area (ha)	Difference (ha)	Difference (%)
S11^b^	20	26,472	5576	20,895.61	−78.9
S14	12	32,454	9426	23,027.38	−71.0
S7	11	9583	3780	5803.16	−60.6
S25	24	28,341	12,192	16,148.12	−57.0
S6	4	8153	3570	4582.34	−56.2
S18	32	29,253	14,054	15,199.08	−52.0
S15	18	30,310	16,236	14,073.42	−46.4
S4	11	19,971	10,858	9113.31	−45.6
S23	21	26,855	15,256	11,598.97	−43.2
S3	14	10,811	6644	4166.75	−38.5
S22	33	17,990	11,106	6883.89	−38.3
S5	17	15,652	10,105	5547.28	−35.4
S13	21	9239	5974	3264.86	−35.3
S12	29	29,726	22,346	7379.34	−24.8
S2	20	14,959	11,341	3618.06	−24.2
S21	17	5937	4922	1014.76	−17.1
S26	29	23,364	19,414	3949.83	−16.9
S24	32	11,154	9820	1334.41	−12.0
S17	6	20,494	19,035	1458.29	−7.1
S27	7	3472	3245	227.60	−6.6
S9	13	5592	5485	107.14	−1.9
Mean	—	18,084.74	10,494.57	7590.17	−42.0

^a^1 ha=10,000 m^2^.

^b^S: subject.

**Figure 1:**
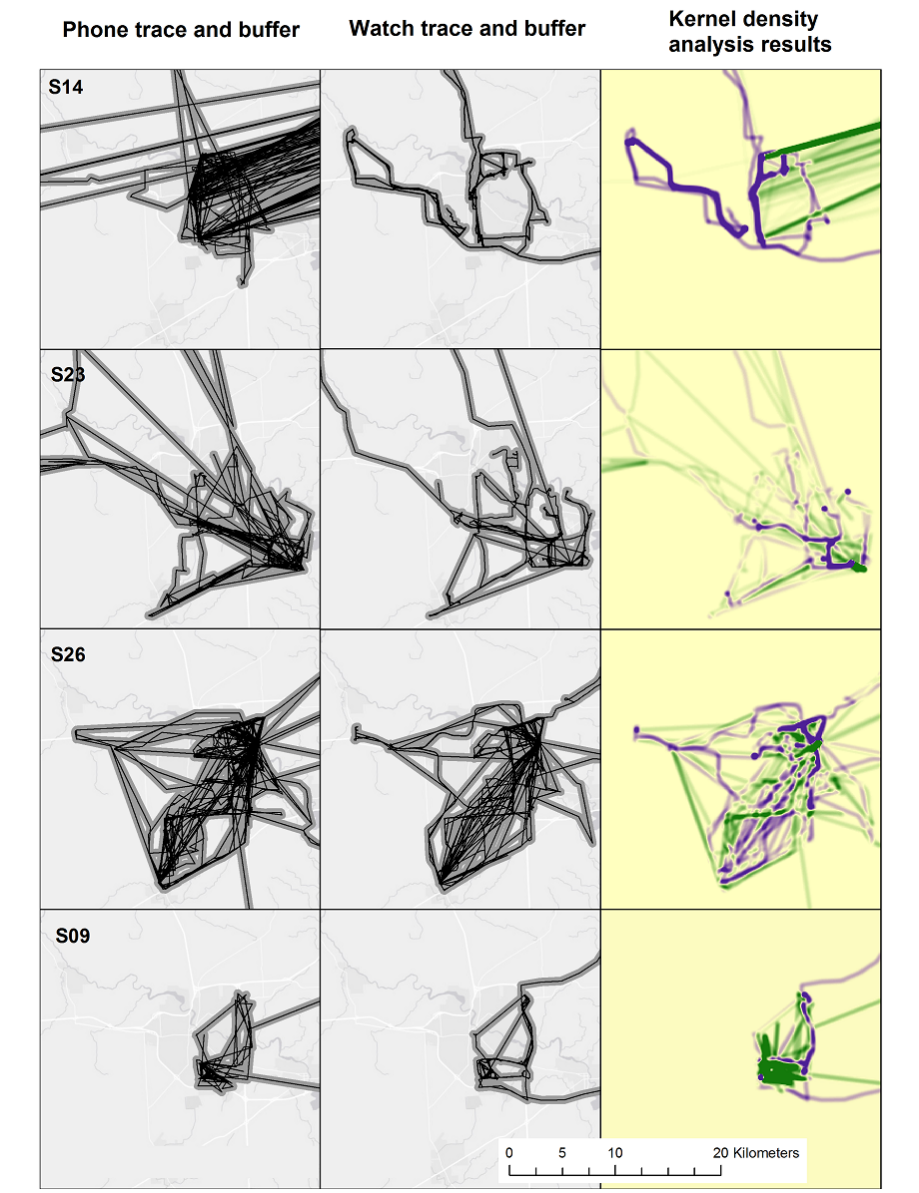
Illustrative activity space data for 4 participants residing in Washtenaw County. The third column shows the difference between the density surfaces computed for the watch and phone data; higher watch density is shown in purple and higher phone density in green. Base map source: Esri [57].

Illustrative examples of this analysis are shown in the first two columns of Figure 1 for participants with extreme (S14 and S23) and minor differences (S26 and S9) in daily paths. To facilitate visual comparison at the same map extent, the 3 participants from outside of Washtenaw County are excluded. This figure illustrates that the watch traces produced activity surfaces with travel closely following highway and street routes. The greater buffer areas produced by the phone are explained by two primary issues: the inclusion of activities in the phone data, which are missing from the watch data, and the phone’s inaccurate representation of highway travel, which resulted in straight lines that artificially inflated the activity space size. The kernel density analysis was conducted to create a more nuanced representation of the differences between the patterns, which can better account for the many overlapping features. The result is shown in the third column of Figure 1, and areas where the phone data resulted in greater density than the watch are shown in green and the reverse pattern in purple. The map can be interpreted as visualizing areas where the participant is more likely to be only because of the choice of device.

**Figure 2:**
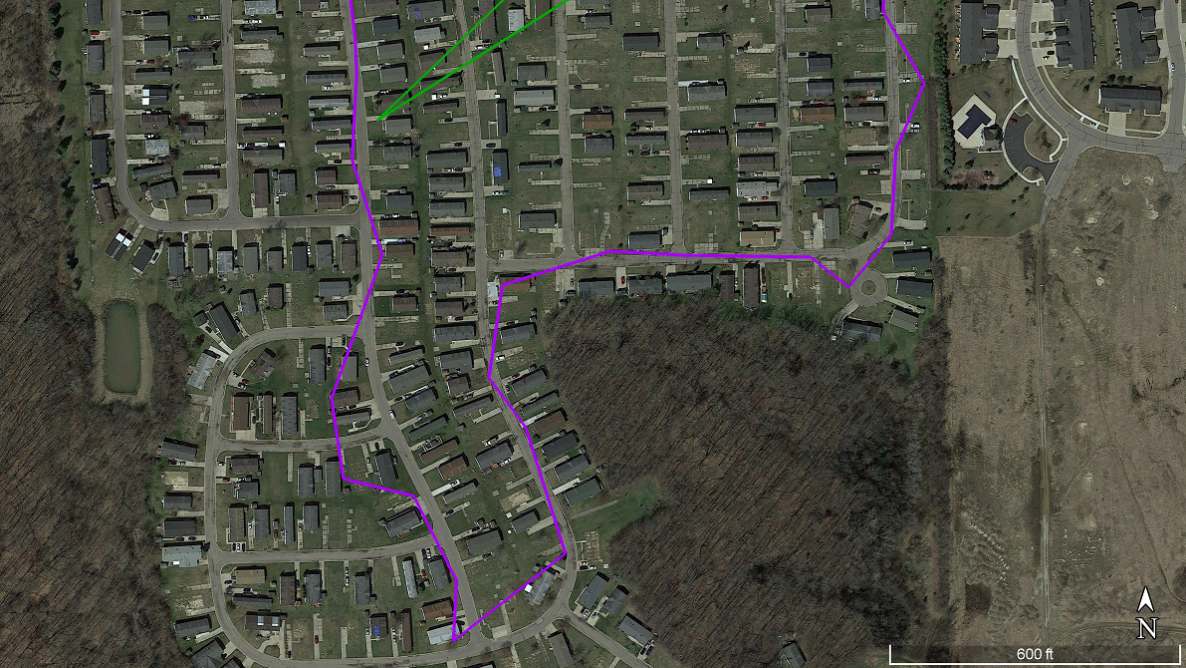
Trace data collected for participant S12 on one study day, illustrating greater spatial detail in watch data (purple) compared with phone data (green) for outdoor exercise (neighborhood walk). Source: Google Earth [58].

The spatial quality of information about outdoor activities, in particular, was higher for the watch than for the phone. For example, as shown in Figure 2, participant S12 described engaging in evening walks near his home, at least one of which was clearly recorded in the watch but not in the phone data.

## Discussion

### Principal Findings

Overall, the phone resulted in a lower proportion of missing data days. In addition, the phone data had a lower mean distance to the known location points and for each of the categories examined, although only the differences in distance to home was statistically significant. The activity space analysis reveals that the phone generated larger activity spaces than the watch. This was because of higher missing data for the watch and inaccurate recording of travel by the phone, which erroneously enlarged the activity space. The watch data resulted in much more accurate data when traveling outdoors, such as riding in an automobile or walking.

### Comparisons With Prior Work

Our research showed that there were activities that were reflected in the phone data but not recorded by the GPS watch. As described in our related work [13], one factor contributing to this difference was user acceptance; as the phone is less obtrusive and it provided other benefits to the participants, they were more likely to carry it. In addition, the participants had difficulty in using the watch, particularly when syncing the device to store data for the study. Unlike the watch that must be synced daily, phone data were logged to a remote server automatically. Although using an alternative GPS device may reduce or eliminate some of these issues, other factors related to the GPS infrastructure, such as the delay in obtaining a satellite signal, would still hinder the widespread use of GPS devices for location tracking. Prior feasibility studies with GPS devices for location tracking in health have surfaced similar issues in use of such devices [32,59]. Moreover, previous work shows that GPS devices typically only function outdoors, where they can connect with multiple satellites, and perform best in places away from dense buildings [10,11].

The spatial accuracy analysis highlights one strength of the phone, which is the improved quality of the collected spatial information about locations in buildings, probably because of the phone’s use of Wi-Fi signals to determine location. Future research could further investigate this issue for specific location categories. Although previous work has shown that a mobile phone–based app can generate accurate spatial data [21], this is the first study, to our knowledge, to empirically compare such devices with other prevailing technologies. Moreover, this study focused on a technology that is available on many mobile phones without requiring the downloading of additional software (Google Location History). The widespread adoption of this technology means that it holds special promise for population-level monitoring and surveillance.

Previous research on the accuracy of location-tracking devices has focused on comparing different types of GPS devices [30,31,60]. This is the first study, to our knowledge, that compares GPS technology with the now-ubiquitous forms of location tracking available in mobile phones. This permitted the identification of sources of error in mobile phone data, which are likely to be increasingly used in research because of their ubiquity.

### Implications for Researchers

The main conclusion we draw from the study is that there are important trade-offs between the use of these two types of devices in health research. Participants successfully collected more data with the phone, with less data loss. In addition, our analysis showed that the phone traces were closer to known destination points than the watch data. However, these benefits came with a significant trade-off, namely, the straight lines and inaccuracies for outdoor activities, described earlier for the phone data. As described earlier, a combination of user behaviors and technical issues explain these results.

Therefore, the suitability of each category of a device depends on the specific research question of a study. Studies that seek precise measurement of outdoor activity and travel, such as measuring outdoor physical activity, characterizing mental health symptoms, or exposure to localized environmental hazards, would benefit from the use of GPS devices. Conversely, studies in which the aim is to account for time within buildings at home or work, or document visits to particular places (such as supermarkets, medical facilities, or fast food restaurants), would benefit from the phone’s demonstrated greater precision in recording indoor activities. For studies that only require the general location and duration of indoor activities, mobile phones may be a more practical data collection device than others that are available to collect this type of information [3]. Furthermore, the weaknesses of each device suggest steps that researchers should take in their research protocols to minimize the associated errors. Given the difficulty of syncing, alternative GPS devices that do not require this step may reduce data losses, if available.

As described, the phone data often included straight-line features that appear to connect points for which detailed locations were obtained by the device. We speculate these lines may be produced through a low data collection rate compared with GPS watches at those times. Consequently, the trace data sometimes included implausible travel routes, which leads us to conclude that the device may have less accurate data for outdoor activities, particularly those that involve travel. Researchers using similar phone data in the future should develop procedures for identifying and removing the straight lines from their datasets. Alternatively, researchers could benefit from the development of a tailored mobile phone app to log location data automatically; this would allow researchers greater control over the nature of the spatial data (such as specifying the desired data collection rate) and allow survey responses to be linked with locations. At the same time, this comes with trade-offs, as these apps will not be adopted at a population level like Google Location History, which could be used to gain new insights into mobility patterns at a large scale.

### Locational Privacy

Although our participants did not express concerns about the privacy of their data, this was primarily because of the use of the data for research purposes, accompanied by our use of ID numbers [13]. Furthermore, in this paper, we included only large-scale maps of the results to minimize the risk of identification [61]. Nevertheless, in general, location tracking has important privacy implications [61]. We acknowledge that studies such as ours raise a variety of issues that, although did not arise here, deserve greater attention from researchers engaging in location tracking in health research studies [33]. These include special considerations for vulnerable populations and what should be done if evidence of harm or illegal activities is observed in participant data [33]. Finally, the paper provides the opportunity to comment on broader privacy issues, as we demonstrate the usefulness of the Google Location History data, which are passively collected for many millions of users. Although Google provides users with the ability to deactivate and delete location history, the sensitivity of the information raises important questions about how this information should be managed in ways that minimize the risk of harm to participants and protect their anonymity and confidentiality.

### Study Limitations

Several limitations of this study should be kept in mind. First, the difficulties that participants faced in using the GPS watch may not persist with newer generations of wearable GPS technology; this may reduce discrepancies in missing data. Second, the straight-line errors concerning travel routes had a differential impact based on the method of characterizing activity space. The buffer methodology, which does not account for the frequency of trips in any particular area, was particularly sensitive to this issue. However, the kernel density analysis somewhat reduced the effect of these features, particularly for participants who collected the most data. Finally, this study was also conducted with a small sample of relatively educated adults in an urban environment, who were recruited through online means. Therefore, user-related data accuracy difficulties may be even greater in populations with less education and technology experience. The generalizability of these results to areas with lower Wi-Fi or cellular tower density (eg, rural areas) is therefore unclear. Finally, although we are unaware of major changes to the technologies here, future improvements to GPS receivers or mobile phone location services may affect the future generalizability of these results.

### Conclusions

Health research increasingly uses fine-grained spatial data gathered from mobile devices to evaluate relationships between health, place, and mobility. Such research, which may be conducted with wearable GPS devices or mobile phones, requires accurate spatial data for analysis. This study reports a direct comparison of the spatial information collected from each of such devices during a field study involving 21 participants. Mobile phones resulted in less missing data, spatial data closer to known destination points, and larger activity spaces. In contrast, the mobile phone data resulted in the recording of outdoor travel inaccurately, including physical activities such as walking. Therefore, the best device for health research depends on the particular study objectives, and data generated from both devices require careful analysis to ensure quality and completeness.

## Abbreviations

EMA: ecological momentary assessment
GPS: global positioning system
PACO: Personal Analysis Companion
